# pH Threshold Impacts Chalcopyrite Bioleaching Dynamics for the Extreme Thermoacidophile *Sulfurisphaera ohwakuensis*

**DOI:** 10.1002/bit.28945

**Published:** 2025-01-31

**Authors:** Daniel J. Willard, Mohammad J. H. Manesh, Kaitlyn M. John, Robert M. Kelly

**Affiliations:** ^1^ Department of Chemical and Biomolecular Engineering North Carolina State University Raleigh North Carolina USA

**Keywords:** archaea, bioleaching, chalcopyrite, extreme thermoacidophiles, *Sulfurisphaera*

## Abstract

The extremely thermoacidophilic archaeon *Sulfurisphaera ohwakuensis* served as the basis for probing how initial pH (pH_initial_) affects copper mobilization from chalcopyrite. Screening of small‐scale cultures (75 mL) at 75°C revealed that ~pH 3.0 was a maximal threshold for bioleaching onset. Subsequently, chalcopyrite at 10 g/L in 750 mL culture media, containing small amounts of ferric ion, adjusted to pH 2.5 with sulfuric acid and incubated for 24 h at 75°C before inoculation, brought the pH to approximately 3.0 through abiotic chemical reactions. However, the resulting subtle differences in pH_initial_ (3.0 ± 0.15) in bioleaching cultures, while not affecting microbial growth, were critical to bioleaching onset and progress. Initial iron levels were less important than pH_initial_ in starting the bioleaching process. X‐Ray Diffraction (XRD) surface analysis informed bioleaching trajectories over 21 days and reinforced the impact of pH_initial_. The subtle differences in pH_initial_ markedly affected *S. ohwakuensis* onset and outcomes, as it presumably would for other bioleaching thermoacidophilic archaea. Furthermore, the findings here highlight the challenges faced in replicating bioleaching experiments across, and even within, laboratories as well as in achieving consistent results in bioleaching processes.

## Graphical abstract

Chalcopyrite bioleaching by the extreme thermoacidophile, *Sulfurisphaera ohwakuensis.*

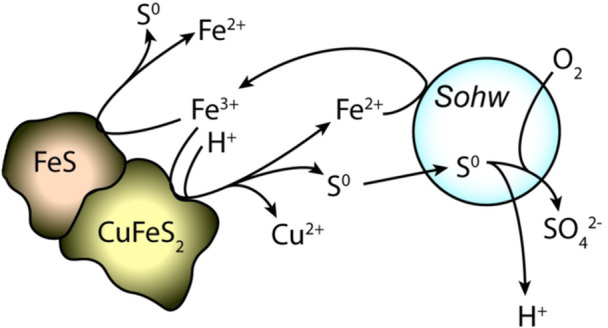

## 1 Introduction

Extremely thermoacidophilic archaea (growth T_opt_ ≥ 65°C, pH_opt_ ≤ 3.5) are indigenous to thermal mining environments and extract bioenergetic benefits from oxidation of reduced iron and sulfur moieties associated with pyritic ores (Manesh et al. 2024). This feature has made them attractive candidates for bioleaching applications (Donati, Castro, and Urbieta 2016). Most prevalent are species from the genera *Sulfolobus*, *Metallosphaera*, *Sulfuracidifex*, *Sulfurisphaera,* and *Acidianus* that are present at varying levels within natural consortia. Metagenomic analysis can determine and track population characteristics within bioleaching consortia, but the respective roles and contributions of individual microorganisms to the mobilization of metals, such as copper from chalcopyrite, are difficult to decipher. Thus, most of what is known about the details of iron and sulfur biooxidation at elevated temperatures and related physiological features come from the study of individual extreme thermoacidophiles in laboratory monocultures. Nevertheless, as more information emerges about the genomes of individual extreme thermoacidophiles (Counts, Willard, and Kelly 2021), microbiological features that define an efficient bioleacher become clearer (Counts, Vitko, and Kelly 2021; Manesh et al. 2024). These insights can then be extended to understanding mechanisms associated with bioleaching consortia (Ríos et al. 2024).

Physical, chemical, and biological phenomena all contribute to the complex nature of bioleaching systems, making it difficult to elucidate specific drivers for metal mobilization. From the biological perspective, cellular metabolism, membrane energetics, biofilm formation, cell motility, metal inhibition, and metal resistance reflect response to environmental conditions (temperature, pH, and redox potential [ORP]), concentration of chemicals (metals, sulfur species, organic carbon, CO_2_, and O_2_), and physical characteristics of the ore body (metal composition and distribution, pulp density, and particle size; Manesh et al. 2024). Unlike laboratory bioleaching studies, ore deposits are open systems in which conditions are not controlled. The challenge is to leverage what is learned in the laboratory to develop better bioleaching processes, whether they are operated in open heaps or in tanking systems.

Several extreme thermoacidophiles have been studied for their ability to mobilize copper from chalcopyrite, including *Sulfuracidifex metallicus* (Gautier, Escobar, and Vargas 2008; Nemati, Lowenadler, and Harrison 2000), *Metallosphaera sedula* (*Msed*; Ai et al. 2019; Auernik and Kelly 2010), and *Acidianus brierleyi* (Dinkla et al. 2009). Optimal bioleaching conditions are specific to each archaeon in this group, where initial pH, Fe^2+^/Fe^3+^ concentration, ORP, and temperature all play a role albeit to varying extents (Vilcáez, Suto, and Inoue 2008). Nevertheless, natural and synthetic thermophilic consortia containing these species and others have been successfully adapted for chalcopyrite bioleaching (d'Hugues et al. 2002; Gericke, Govender, and Pinches 2010; Gericke, Pinches, and Van Rooyen 2001; Li et al. 2014; Mikkelsen et al. 2006). For example, advantages with high‐temperature bioleaching were demonstrated in a pilot‐scale tanks‐in‐series system that successfully bioleached low‐grade nickel‐Cu concentrates using a consortium containing *Acidianus*, *Metallosphaera*, and *Sulfuracidifex* species; however, the contributions of specific microbes were not determined (Neale 2009). Similar findings came from bioleaching studies with a chalcocite‐dominant Cu ore from Salta, Argentina, where indigenous thermophiles (including *Acidianus copahuensis* and *Ferroplasma* sp.) outperformed less thermophilic microbes (Amar et al. 2023). These promising results motivate continued efforts to develop high‐temperature bioleaching processes.

As more is learned about the physiology, ecology, and metabolism of extreme thermoacidophiles for the development of high‐temperature bioleaching processes (Manesh et al. 2024), details of the complex ways in which physical, chemical, and microbiological phenomena factor in the need to be understood. This will help in choosing the best operating conditions and microbes (monocultures or consortia) for optimal results. However, the intrinsic kinetic and thermodynamic properties characteristic of bioleaching systems can make it difficult to replicate and reproduce results, not only across laboratories but also within laboratories. Certainly, reports of bioleaching studies are often based on a single replicate for this reason (Córdoba et al. 2008; Ranjbar et al. 2017; Vilcáez, Suto, and Inoue 2008; Zhao et al. 2017; Zhao et al. 2014). Here, the extremely thermoacidophilic archaeon, *Sulfurisphaera ohwakuensis* (*Sohw*), served as the basis for examining the dynamics and trajectory of chalcopyrite bioleaching with an eye toward discerning how subtle differences in experimental conditions can impact experimental outcomes and skew interpretation of results.

## 2 Materials and Methods

### 2.1 Microorganisms Studied and Bioleaching Cultivation Conditions

*Sohw* (DSM‐12421) was obtained from the Leibnitz Institute DSMZ (Braunschweig, Germany) and grown at 75°C in monoculture using appropriate media and conditions. Growth media was formulated as follows: Brock's Salts (DSM‐88 medium): 1.30 g/L (NH_4_)_2_SO_4_, 0.28 g/L KH_2_PO_4_, 0.25 g/L MgSO_4_·7H_2_O, 0.07 g/L CaCl_2_·2H_2_O, 0.02 g/L FeCl_3_·6H_2_O, 4.5 mg/L Na_2_B_4_O_7_·10H_2_O, 1.8 mg/L MnCl_2_·4H_2_O, 0.22 mg/L ZnSO_4_·7H_2_O, 0.22 mg/L Na_2_MoO_4_·2H_2_O, 0.05 mg/L CuCl_2_·2H_2_O, 0.03 mg/L VOSO_4_·2H_2_O, and 0.01 mg/L CoSO_4_·7H_2_O; sulfuric acid was used to adjust the pH of the medium. *Sohw* is a heterotrophic microorganism; therefore, cultures also included 1 g/L yeast extract as a nutrient source. All cultures were sealed with foam stoppers. All chalcopyrite bioleaching experiments were inoculated at a starting OD_600_ of 0.01 (~10^7^ cells/mL). Small‐scale screening cultures were grown on 10 g/L chalcopyrite in 75 mL in 150 mL serum bottles, inoculated 3 h after adjusting the pH_initial_ to 2.0, 2.5, 3.0, or 3.5. To investigate the effect of pH further, three larger‐scale cultures were grown on 10 g/L chalcopyrite at 750 mL in 1 L Erlenmeyer flasks following incubation of chalcopyrite for 24 h before inoculation. Despite efforts to replicate experimental conditions, at the time of inoculation, the pH in the three flasks had varied slightly (2.94, 3.03, or 3.15) likely due to subtle differences in rates of abiotic chemical reactions. Note that the ORION STAR A211 pH Meter and probe (Thermo Fisher Scientific, Massachusetts, the United States) used were capable of measuring pH with an accuracy of ± 0.01 units.

### 2.2 Chalcopyrite Bioleaching Experiments

Chalcopyrite (BOC Sciences, New York, the United States) samples were obtained at 325 mesh pass rates ≥ 97% (dry brush method). Pulp density across all experimental bottles was 10 g/L, at 75°C, and sampling took place immediately after inoculation and on Days 5, 15, and 21 for the 75 mL cultures. For 750 mL cultures, sampling was done every 24 h until Day 5, every ~12 h until Day 15, and then every 3 days until Day 21. For sampling, 2 mL of 75 mL cultures were used on a magnetic rack to separate the chalcopyrite, and the remainder was used initially for OD_600_ measurements, sterile filtered using 0.22‐μm syringe filters, and stored at −20°C for follow‐up measurements. For 750 mL cultures, 80 μL was used separately for counting cells using acridine orange staining and epifluorescence microscopy, and 4 mL of cultures were centrifuged briefly, after which the supernatant was filtered as mentioned above and stored for follow‐up measurements.

Measurements at each timepoint included ORP (PINPOINT ORP Monitor [American Marine Inc., Connecticut, the United States]), pH (ORION STAR A211 pH Meter [Thermo Fisher Scientific], total iron and ferrous iron concentration (1,10‐phenanthroline method [Manesh et al. 2024], copper concentration (bis[cyclohexanone] oxaldihydrazone method [Manesh et al. 2024]), and sulfate concentration (barium sulfate method [Zeldes et al. 2019]). A Biotek Cytation 5 Imaging Reader (Agilent, California, the United States) was used to measure absorbances for iron, copper, and sulfate measurements.

### 2.3 Mineral Surface Analysis

Residual solids from sampling at each timepoint for the 750 mL cultures were washed once with Brock's Salts at pH 2.5, once with distilled water, dried overnight at 75°C, and stored for X‐Ray Diffraction (XRD) analysis. Endpoint solids after bioleaching were similarly prepared for the 75 mL cultures. All three starting pH values of the 750 mL cultures and the cultures with the highest copper extraction from the 75 mL cultures were selected for XRD analysis. A SmartLab XRD (Rigaku, the United States) was used for XRD analysis. HighScore Plus software was used for data analysis and phase recognition, and the matching peak scores were normalized and used to represent a semi‐quantitative representation of the XRD results.

## 3 Results and Discussion

### 3.1 Chalcopyrite Bioleaching by *Sohw*

*Sohw* (Kurosawa et al. 1998), originally isolated from muddy waters (70–80°C) in acidic hot springs ( ~pH 3) in Hakone, Japan, outperformed other previously studied bioleaching extreme thermoacidophiles available from culture collections for mobilizing copper from chalcopyrite (Manesh et al. 2024). *Sohw* is a facultatively anaerobic organotroph that can oxidize elemental sulfur. Its genome encodes the Fox cluster (Counts, Vitko, and Kelly 2021; Manesh et al. 2024), indicating that it should also be capable of iron oxidation. While bioleaching survey results can vary from lab to lab, the identification of *Sohw* as an efficient bioleacher was unexpected, given that it has not been studied in any detail previously. That being said, a strain related to *Sohw* was the main component of an adapted bioleaching consortia operated at 78°C and pH 1.6 with high copper recoveries (Mikkelsen et al. 2006). Thus, *Sohw* was selected here for further analysis to determine the dynamics and trajectory of the chalcopyrite bioleaching process.

### 3.2 Initial pH Impacts Copper Release by *Sohw* (75 mL Scale)

Previous work in our lab showed that for chalcopyrite bioleaching with several extreme thermoacidophiles pH 3 seemed to be a threshold for initiating and driving copper release (Manesh et al. 2024; Manesh et al. 2024). Although pH can be controlled in small‐scale laboratory bioleaching studies, this will be more challenging at larger scales where pH can vary as microbial action and ore chemistry conflate. Thus, the key at larger scales will be to maintain pH in a range that supports bioleaching. Here, while *Sohw* growth was not affected as pH varied slightly above or below at a pH of 3, bioleaching was impacted to a significant extent. This sensitivity of bioleaching to pH was not observed at lower pH values. Thus, pH of 3 was investigated as a threshold for bioleaching performance for *Sohw* and potentially a concern for other extreme thermoacidophiles.

**Figure 1:**
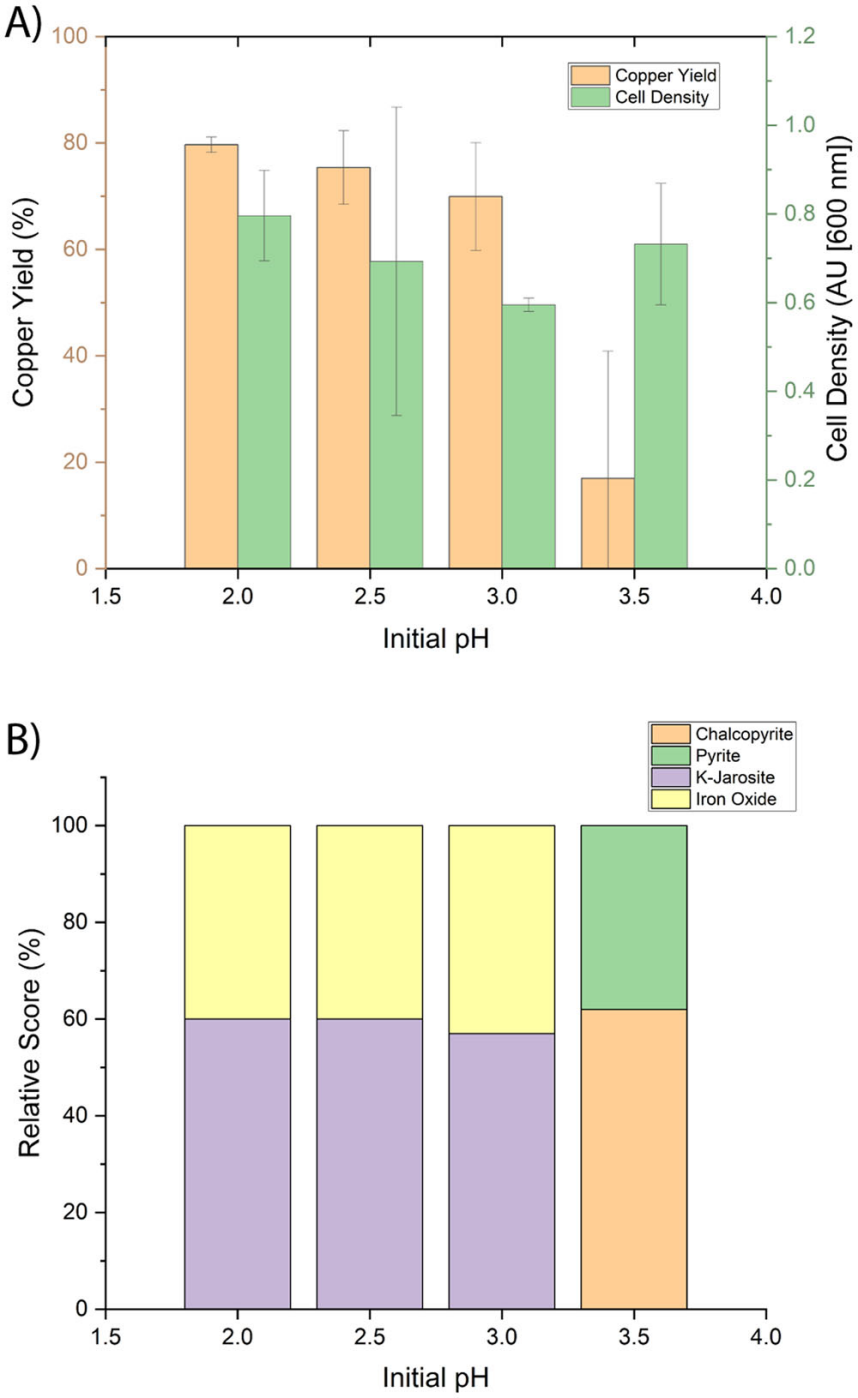
(Top) Cell density and fraction of copper mobilized after 21 days as a function of pH_initial_. While cell growth was significant for all initial pH values, only limited amounts of copper were mobilized with a starting pH of 3.5. (Bottom) XRD surface analysis following 21 days of bioleaching. For an initial pH of 2 or 2.5, the surface was mostly covered with iron oxide and ammonium jarosite. At pH 3, some chalcopyrite was also present. At pH 3.5, more chalcopyrite was present, as was pyrite. The presence of chalcopyrite indicated that the bioleaching process was in its early stages, as reflected in copper release.

Bioleaching with *Sohw* was screened in 75 mL cultures to investigate the impact of pH_initial_ on bioleaching performance. Chalcopyrite was allowed to incubate for 3 h before inoculating with *Sohw*. After 21 days, copper solubilization reached up to 80% yield for cultures with initial pH_initial_ ≤ 3.0, while limited amounts of copper were mobilized from cultures with a pH_initial_ of 3.5 (Figure 1). Notably, the variability in copper solubilization between replicates at each pH_initial_ decreased significantly as pH decreased, demonstrating that, while *Sohw* is capable of efficient bioleaching at a pH_initial_ up to ~3.0, the dissolution of chalcopyrite is more pronounced at lower pH_initial_. This indicated that the chemical environment in the early stages of bioleaching had a significant impact on the onset and ultimate trajectory of a bioleaching system. Despite the variability in bioleaching outcomes, the cell density of the cultures was consistent across the pH_initial_ range explored, even in cases where copper yield was very poor. Thus, the catalytic role of *Sohw* in a bioleaching system was not the sole determining factor influencing the ultimate bioleaching outcome.

XRD surface analysis was performed on the 75 mL samples following 21 days of bioleaching. For pH_initial_ of 2, 2.5, or 3.0, the mineral surface was mostly covered with iron oxide and potassium jarosite, both expected byproducts of the bioleaching process. At pH_initial_ 3.5, chalcopyrite and pyrite were evident, indicating that the bioleaching process was still in the early stages (also reflected by low levels of copper release). Thus, the pH_initial_ 3.5 condition did not necessarily stall out during the bioleaching process but rather had a significant kinetic lag compared to the lower pH_initial_ conditions.

**Figure 2:**
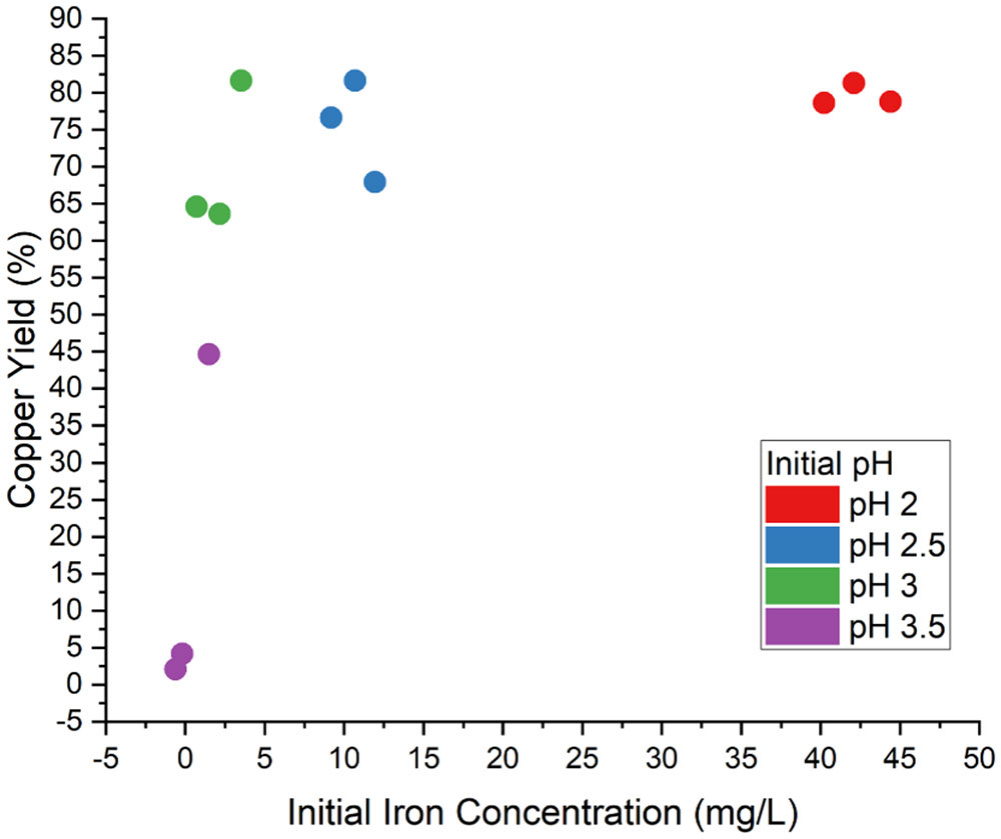
Variability of initial iron concentration of different starting pH bioelaching cultures to the eventual copper yield. Above 1 mg/L, initial ferric iron does not significantly increase the copper yield. However, the presence of iron above the threshold is required for the initiation of bioleaching.

Iron concentrations at the time of inoculation exhibited significant variation among pH values, ranging from < 1 mg/L at pH_initial_ 3.5 to 40–45 mg/L at pH_initial_ 2.0, despite all media being prepared with an initial concentration of 4.1 mg/L ferric iron (Figure 2). Thus, the variability in iron concentration was attributed to the 3 h abiotic incubation period before inoculation of *Sohw*. At pH_initial_ 3.5, the decrease in iron concentration during abiotic incubation, likely as a result of precipitation, may explain the poor bioleaching outcomes due to a lack of Fe^3+^ to initiate the attack on the chalcopyrite. In one pH_initial_ 3.5 replicate, the iron concentration remained > 1 mg/L, and moderate copper solubilization was observed. However, the pH_initial_ 3.0 cultures exhibited a similar iron concentration to the pH_initial_ 3.5 replicate and vastly outperformed it. Indeed, above the 1 mg/L threshold, the initial iron concentration appeared to have little influence on the ultimate copper yield and is arguably more a consequence of the effect of pH_initial_ on abiotic solubilization of chalcopyrite.

### 3.3 Early Bioleaching Conditions Affect the Trajectory of Copper Release

**Figure 3:**
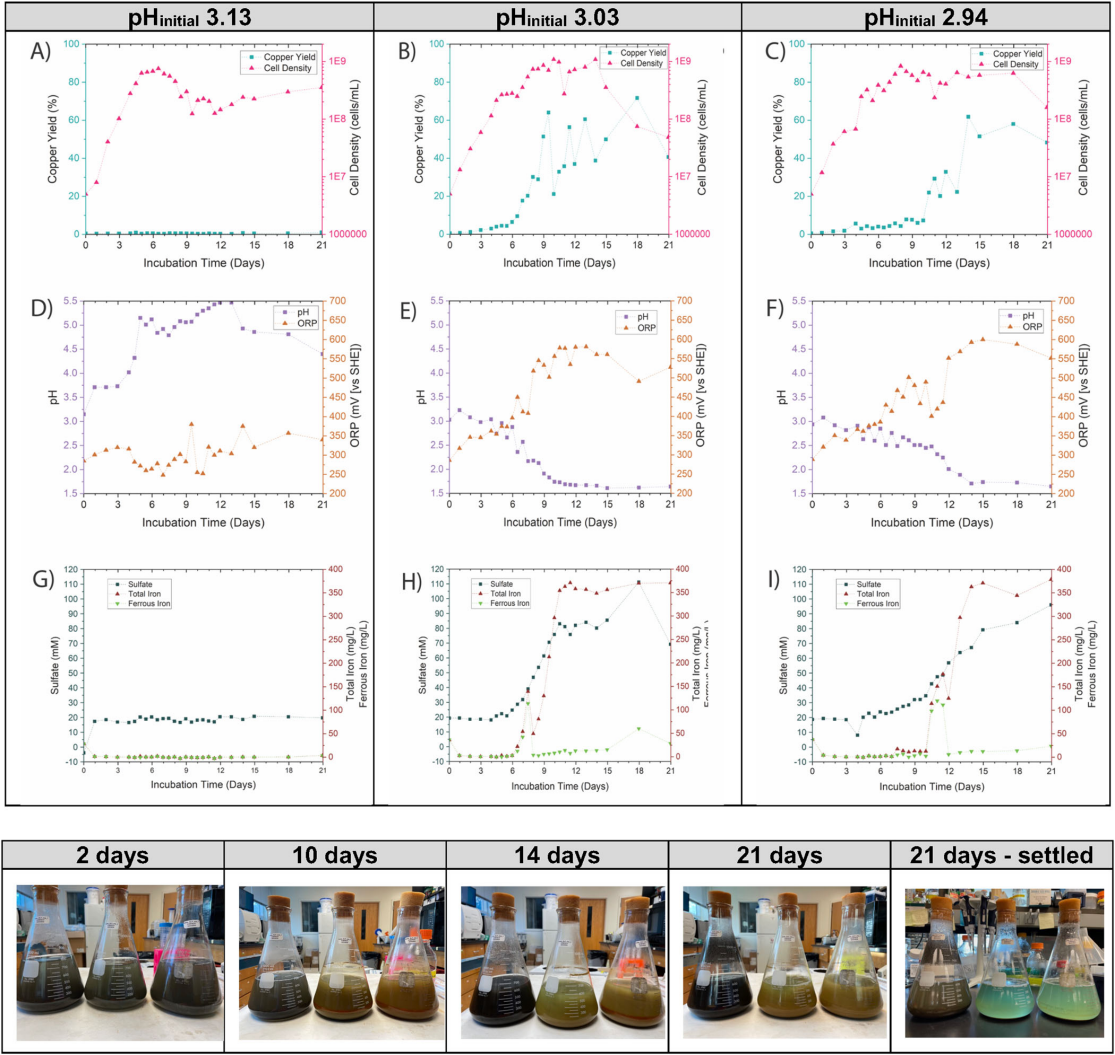
Chalcopyrite bioleaching cultures (750 mL) were inoculated with *S. ohwakuensis* at pH_initial_ 3.15, 3.03, and 2.94 at 75°C followed over 21 days. (Top) (A–C) Copper % yield (cyan) and cell density (magenta); (D–F) pH (purple) and redox potential (orange); (G–I) total sulfate concentration (blue), total iron concentration (red), and ferrous iron concentration (green). (Bottom) Cultures (750 mL) (left–right: 3.15, 3.03, and 2.94) at various points over 21 days of bioleaching. Note that 504 h culture shown following 60 min of settling reveals azure color, indicating copper solubilization.

Based on the results from the 75 mL scale cultures, larger‐scale (750 mL) experiments were done centered around pH 3 to further understand the influence of pH_initial_ on the onset and trajectory of bioleaching. Cultures were prepared at pH 2.5 and allowed to incubate abiotically for 24 h before inoculation to allow any chemical changes to equilibrate. Bioleaching experiments can be difficult to replicate exactly, given the interaction of physical, chemical, and biological phenomena, and this was also noted here. At the point of inoculation, pH had varied slightly among the replicates reflecting small differences in mixing, aeration, and mineral characteristics, among other factors, such that the pH of cultures had shifted slightly to 2.94, 3.03, or 3.15. Over the course of 21 days of bioleaching, *Sohw* grew comparably for all three pH_initial_ values, based on measurement of planktonic cell OD_600_. However, for pH_initial_ 3.15, there were no signs of bioleaching, while for pH_initial_ 3.03 and pH_initial_ 2.94, 72% and 62% copper yield were observed, respectively (Figure 3A–C). Notably, the onset of copper release from the pH_initial_ 3.03 and pH_initial_ 2.94 conditions at the 750 mL scale lagged behind the copper release of the 75 mL scale pH_initial_ 3.0, which reached 15%–25% copper yield by Day 5. Despite this, the 750 mL cultures ultimately reached comparable endpoint copper yield to the 75 mL scale, indicating there may be some scale‐up effects on the kinetics of bioleaching perhaps related to solids distribution and O_2_ mass transfer, among other factors.

Both pH_initial_ 2.94 and pH_initial_ 3.03 cultures saw a small increase in pH at 24 h after inoculation but exhibited steady acidification beyond that point. One contributing factor is the precipitation of iron ions as jarosite, which leads to acidification. In contrast, the pH_initial_ 3.15 culture saw an increase in pH up to 3.71 at 24 h, where it held stable for 2 days before neutralizing further to pH > 5 (Figure 3D–F). It is unclear if this dramatic proton consumption is linked to some small amount of ore dissolution or that the lack of available sulfur to be oxidized caused *Sohw* to consume protons to support its growth; likely, it is some combination of both.

ORP started between 285 and 290 mV (vs. SHE) and increased to 300–320 mV after 24 h for all three conditions. At pH_initial_ 3.15, ORP remained constant at this point, averaging 300 mV and never exceeding 380 mV over the course of the entire 21 days. For pH pH_initial_ 2.94 and pH_initial_ 3.03, the cultures exhibited a continuous increase in ORP to > 400 mV over the course of the first 6 days (Figure 3G–I). Both cultures then exhibited a rapid jump in ORP to > 500 mV before stabilizing around 550 mV; note that this occurred at Day 8 for pH_initial_ 3.03 and at Day 12 for pH_initial_ 2.94. Favorable ORPs with appropriate concentrations of Fe^2+^ have been reported as critical, since low ORPs (350–450) favor Fe^2+^ over Fe^3+^, leading to less passivation and more leaching efficiency (Tian et al. 2021).

Sulfate concentration was an indicator of sulfur oxidation for reduced inorganic sulfur compounds (RISCs) by *Sohw*. For pH_initial_ 3.15, little to no sulfate production was observed beyond 24 h, which is not surprising since chalcopyrite dissolution is the source of RISCs in this system and minimal chalcopyrite dissolution occurred in this case. For pH_initial_ 2.94 and pH_initial_ 3.03, sulfate concentrations remained constant until Day 4, after which a rapid and steady increase in sulfate was detected for the remainder of the 21 days (Figure 3). Biological oxidation of sulfur and RISCs acts as the source of protons in bioleaching systems, so it is notable that acidification of the pH_initial_ 2.94 and pH_initial_ 3.03 conditions preceded the detectable increase in sulfate concentration. Only small amounts of sulfur and RISCs would be available in the system during early bioleaching, so the amount of sulfate produced before Day 4 was likely below the detection limit, despite having a noticeable impact on pH. Another possibility, though, is that sulfate precipitation was occurring even during early bioleaching, and the increasing sulfate concentration was only detectable once sulfur oxidation rates significantly increased after Day 4. This is consistent with the observation that soluble iron concentrations dropped to near zero during the first 24 h of leaching for all three pH_initial_ (Figure 3). No appreciable soluble iron was detected at any point in the pH_initial_ 3.15 case, and iron concentrations did not increase the pH_initial_ 2.94 and 3.03 conditions until after the ORP exceeded 400 mV. At this threshold, a rapid spike in both total iron and Fe^2+^ occurred; total iron continued to increase in conjunction with the rise in sulfate concentration, while Fe^2+^ concentrations rapidly dropped to very low levels for the remainder of the 21 days.

The timing of events around the iron spike plays a key role in parsing out the mechanisms of action involved in the *Sohw* bioleaching system. The increase in ORP of bioleaching systems is frequently attributed to the Fe^3+^/Fe^2+^ equilibrium as more iron is solubilized during bioleaching. This appears to be the case for the rapid jump in ORP from 400 mV to > 500 mV for pH_initial_ 2.94 and 3.03 cultures; the spike in iron immediately preceded the ORP increase, and the shift in Fe^2+^/Fe^3+^ ratio coincides with the ORP increase. This is certainly where the bulk of chalcopyrite oxidation occurred, based on the rapid increases in soluble iron, sulfate, and copper around this point. This result is consistent with the reduction potential of chalcopyrite oxidation of 400–440 mV (Manesh et al. 2024). However, the initial steady climb of ORP from 300 mV to > 400 mV occurred well before any signs of soluble iron were present in the system, and chalcopyrite oxidation is thermodynamically unfavorable in these early stages. Thus, an ORP of 400 mV appears to be a critical threshold to enable effective bioleaching by chalcopyrite oxidation. This is further supported by the lack of chalcopyrite dissolution for pH_initial_ 3.15, where the ORP failed to reach 400 mV. Heterogenous oxidation of ferrous iron to ferric iron under acidic conditions is enhanced by iron oxyhydroxides that alter the Fe^2+^/Fe^3+^ ratio and contribute to the availability of ferric iron for bioleaching (Jones et al. 2014).

In spite of this observation, small increases in soluble sulfate and copper were observed in both pH_initial_ 2.94 and 3.03 cases before ORP reached the 400 mV threshold and the appearance of soluble iron. This indicated that dissolution of the ore was occurring by a mechanism other than chalcopyrite oxidation in the early stages of *Sohw* bioleaching.

### 3.4 XRD Analysis of the Mineral Surface During Bioleaching

**Figure 4:**
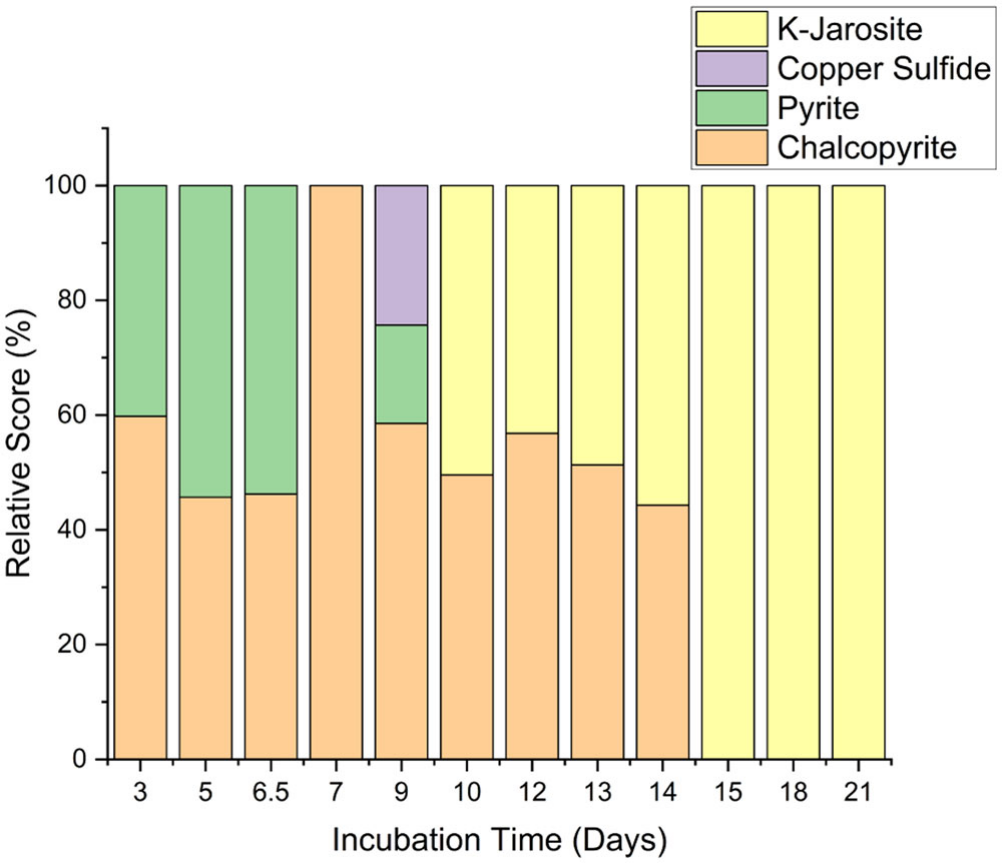
XRD profile of the solid phase during bioleaching at pH_initial_ 3.03. The early disappearance of pyrite (green) and formation of copper sulfide (purple) indicate progressive stages of bioleaching, in which chalcopyrite (orange) is only substantially leached during the later stages of bioleaching and after jarosite (yellow) formation has already begun to occur. Ultimately, coverage of the mineral surface by jarosite appears to terminate the bioleaching process.

To further examine the behavior of the *Sohw* bioleaching during the early leaching stages and to assess the effect of passivation of the ore surface during late‐stage bioleaching, solid samples from the fastest leaching condition (pH_initial_ 3.03) were analyzed by XRD to detect changes to the mineral surface (Figure 4). Initially, the main phases comprising the mineral were chalcopyrite and pyrite, where most of the changes were on the pyrite portion of the mineral. With the increase in total iron present in the culture, by the 167 h timepoint no pyrite was detected, indicating that the initial iron increase was due to iron leaching from pyrite. This also provides more ferrous iron to be converted to ferric iron through biological activity, enhancing copper extraction. Around 191 h, the formation of secondary phases was detected, mainly copper sulfides and iron sulfides in the form of pyrite. With the significant increase in iron and sulfur solubilization, the formation of passivating layers, mainly potassium jarosite, was then detected throughout the bioleaching experiment. By the 359 h timepoint, all the remaining chalcopyrite is masked by the passivating layers on the mineral surface. *Sohw* alleviated the formation of passivating layers of precipitates via its strong sulfur oxidation capability, leading to high levels of copper extraction, even compared to other thermoacidophiles (Manesh et al. 2024). However, chemical reactions related to potassium jarosite formation outpace sulfur solubilization by *Sohw* after a certain timepoint, leading to the effective termination of copper solubilization. This happened at about Days 9 and 10 for the cultures that had significant copper extraction.

Therefore, another way by which *Sohw* can be more effective than other thermoacidophiles is by its ability to extend the effective bioleaching time by alleviating the passivation problem for prolonged periods. *Msed*, for example, has been shown to have a faster initial copper release, but its bioleaching efficacy is hindered soon thereafter by surface passivation (Manesh et al. 2024). Since *Msed* is known to be an efficient iron oxidizer (Counts, Vitko, and Kelly 2021), this contributes to jarosite formation without any counterbalancing action.

### 3.5 Chalcopyrite Bioleaching by *Sohw* Progresses Through Phases

**Figure 5:**
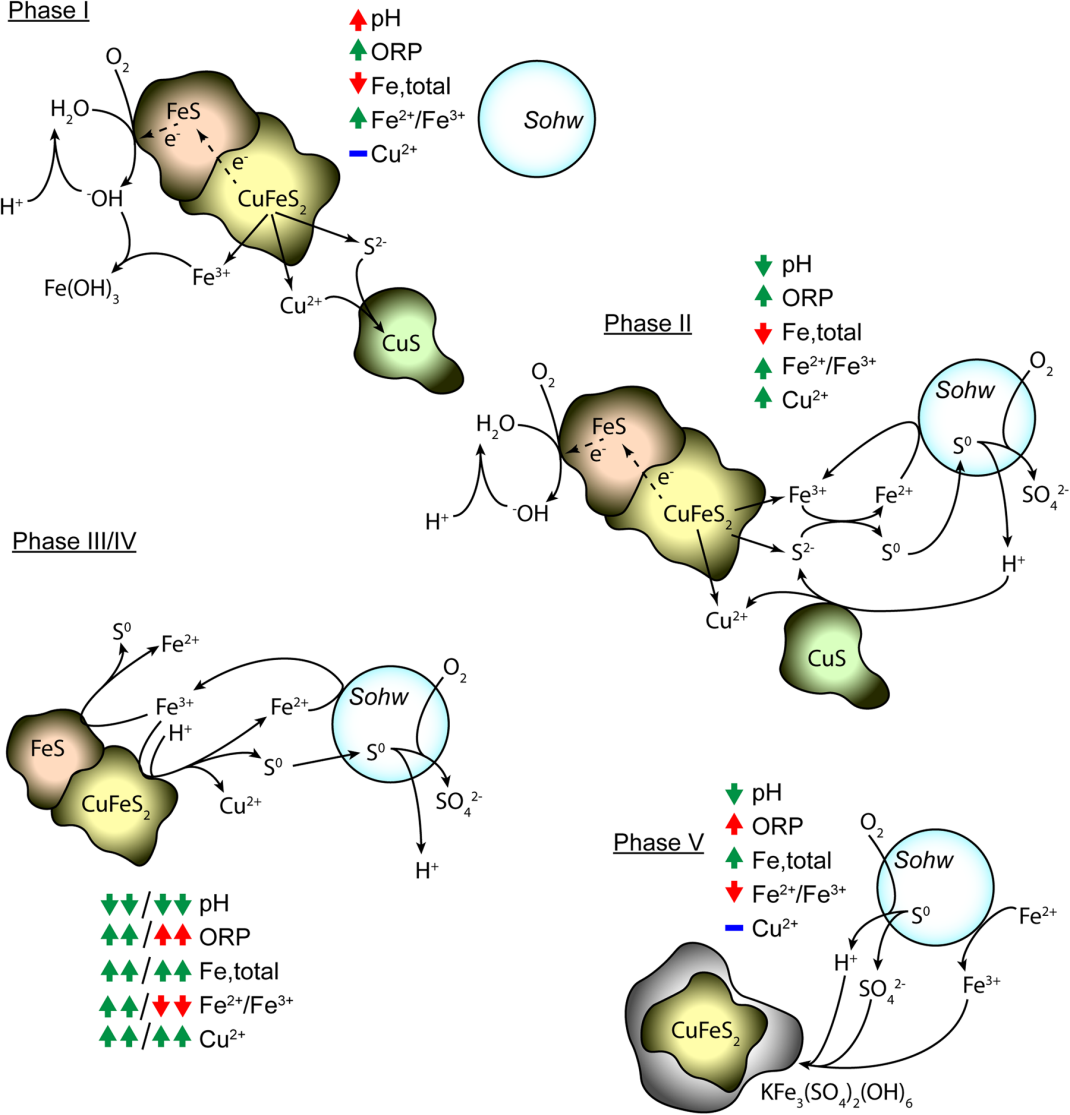
Progression of *Sohw* chalcopyrite bioleaching progresses through phases. *Phase I:* oxygen‐dependent galvanic interaction between chalcopyrite and pyrite leads to the initial release of sulfide, with iron and copper precipitating due to less acidic conditions. *Phase II: Sohw* oxidizes sulfide and elemental sulfur to sulfate, generating protons and beginning to solubilize copper sulfide intermediates. *Phase III*: decreasing pH and soluble copper drive an increase in redox potential (ORP), allowing oxidative bioleaching to start breaking down pyrite and chalcopyrite in earnest. *Phase IV:* once the ORP exceeds ~450 mV, the ferrous‐to‐ferric iron ratio shifts and most iron exists in the form of ferric iron, causing extremely rapid chalcopyrite dissolution. *Phase V:* rapid increases in ferric iron lead to the precipitation of jarosite compounds, which cover the ore surface and prevent further dissolution of chalcopyrite.

Based on the bioleaching trajectory of *Sohw*, the onset of chalcopyrite bioleaching proceeds through several phases (see Figure 5). Initially, the low ORP of the system is unfavorable for chalcopyrite oxidation (*Phase 1*). However, galvanic action between pyrite and chalcopyrite consumes protons through the reduction of O_2_ and can drive the release of small amounts of copper and sulfide, leading to the formation of a copper sulfide phase. Iron released from chalcopyrite during this process forms ferric hydroxide, which is highly insoluble in water but becomes more soluble under more acidic conditions. These events result in a stoichiometric excess of sulfur and RISCs such that some sulfur remains in solution to be oxidized by *Sohw* to offset the neutralizing effect of galvanic activity.

During *Phase 2* of bioleaching, the rate of proton generation from *Sohw* sulfur oxidation outpaces the rate of proton consumption, and the system begins to acidify. As the pH decreases, copper sulfide and ferric hydroxide begin to dissolve back into the solution. Copper can affect the ORP of bioleaching systems, and small amounts of soluble copper in early bioleaching stages may drive the increase in ORP to > 400 mV.

Clearing the 400 mV ORP threshold represents the transition to *Phase 3* of bioleaching, where chalcopyrite oxidation becomes favorable. The solubilized ferric hydroxide supplies Fe^3+^ ions to facilitate this reaction, which are quickly reduced to Fe^2+^ by chalcopyrite. The process becomes limited by the ability of *Sohw* to oxidize Fe^2+^ ions and regenerate the Fe^3+^ pool. The release of significant amounts of iron and copper drives the ORP up above 500 mV, triggering *Phase 4* of bioleaching. At this point, the Fe^2+^/Fe^3+^ equilibrium shifts to favor Fe^3+^, and biological iron recycling is no longer a rate‐limiting step. Chalcopyrite oxidation occurs rapidly, and most of the copper is solubilized during this phase.

Ultimately, the ORP climbs high enough to favor the precipitation of jarosite compounds. This represents *Phase 5* and the final phase of bioleaching, where the mineral surface becomes covered in jarosite, blocking attack by Fe^3+^ and protons and thereby passivating the mineral. At this point, minimal gains in copper yield occur and bioleaching has been effectively terminated.

In chalcopyrite (bio)leaching, the properties of the mineral itself also need to be taken into account. Specifically, the semiconductor properties of chalcopyrite limit the current flow until a breakpoint is reached, but it is inhibited by the presence of impurities, making it pseudo‐metallic (O'Connor and Eksteen 2020). These properties, alongside the operating conditions of chalcopyrite (bio)leaching affect the copper recovery efficiency. While passivation as a hindrance to chalcopyrite dissolution has been controversial (O'Connor and Eksteen 2020), recent studies still highlight passivation as one of the main reasons for partial copper extraction from chalcopyrite (bio)leaching (Sun et al. 2021; Wang et al. 2021), with high temperatures associated with extreme thermoacidophiles suitable for alleviating the adverse effects of aforementioned parameters for copper extraction from chalcopyrite even with passivation occurring (Zhu et al. 2011).

Whether this sequence of events applies to other bioleaching systems involving extreme thermoacidophiles remains to be seen. However, the paradigm discussed here can serve as a reference point for other high‐temperature (or maybe less thermophilic) bioleaching systems, either in monoculture or involving consortia.

## 4 Conclusions

The onset of *Sohw* chalcopyrite bioleaching was especially sensitive to pH, with a pH of ~3.0 representing a threshold for initiating the process. While *Sohw* has been less studied as a bioleacher, its genome suggests that it has many of the same metabolic characteristics as other extreme thermoacidophiles (i.e., sulfur and iron oxidation capability). It is not clear how pH_initial_ impacts bioleaching by natural or synthetic consortia, but pH could serve as a driver in initiating and regulating processes in tanking systems. The results here also illustrate why bioleaching experiments can be difficult to replicate between laboratories and even within the same laboratory, given the sensitivity to the initial chemical environment in these complex systems. This is not surprising given the heterogeneous nature of bioleaching environments, exacerbated here by elevated temperature, for extreme thermophiles.

## Author Contributions

Conceptualization: Daniel J. Willard, Mohammad J. H. Manesh, and Robert M. Kelly. Data curation: Daniel J. Willard and Mohammad J. H. Manesh. Formal analysis: Daniel J. Willard, Mohammad J. H. Manesh, and Robert M. Kelly. Investigation: Daniel J. Willard, Mohammad J. H. Manesh, Kaitlyn M. John, and Robert M. Kelly. Methodology: Daniel J. Willard, Mohammad J. H. Manesh, and Robert M. Kelly. Funding acquisition: Robert M. Kelly. Resources: Robert M. Kelly. Supervision: Robert M. Kelly. Visualization: Daniel J. Willard, Mohammad J. H. Manesh, and Robert M. Kelly. Writing–original draft: Daniel J. Willard, Mohammad J. H. Manesh, and Robert M. Kelly. Writing–review and editing: Daniel J. Willard, Mohammad J. H. Manesh, Kaitlyn M. John, and Robert M. Kelly. All authors contributed to writing and editing the manuscript.

## Conflicts of Interest

The authors declare no conflicts of interest.

## Data Availability

Data will be made available on request.
